# Supplementary material for: Enhanced ZBTB16 Levels by Progestin-Only Contraceptives Induces Decidualization and Inflammation
Source: Int J Mol Sci. 2023 Jun 23;24(13):10532. doi: 10.3390/ijms241310532 (PMC10341894; doi:10.3390/ijms241310532)
Supplement: Supplementary file 1 [file ijms-24-10532-s001.zip › ijms-2366590-supplementary.pdf]

Figure S1. Increased *PRL* and *IGFBP1* expression during *in vitro* decidualization of cultured HESCs. qPCR analysis indicated enhanced expression of decidualization markers, prolactin (*PRL*) and insulin like growth factor binding protein 1 (*IGFBP1*) mRNA levels in HESCs during decidualization at 0, 3, or 6 days following 10^{-8} M E_2+10^{-7} MPA + $5x10^{-5}$ cAMP (EMC) treatment. Data represents mean± SEM; n=5/each; *$p<0.05$ *vs.* Day 0 analyzed by one way ANOVA followed by Student-Newman-Keuls Method.

Supplementary Figure S1

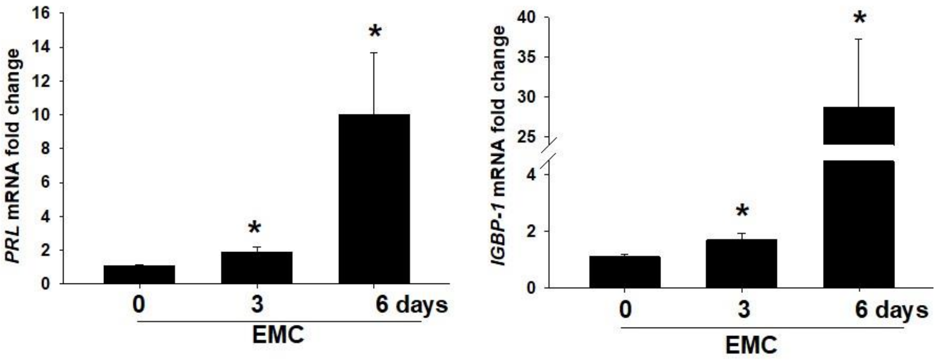

Figure S2. Elevated *ZBTB16* mRNA levels in primary cultured HEECs treated with pLARCs. *ZBTB16* mRNA levels were analyzed by qPCR in HEECs treated with 10^{-8} M E_2 alone as a control or ± 10^{-7} M ORG, or ETO, or LNG, or MPA, or DEX for 7 days. Bars represent mean± SEM; n=3; *$p<0.05$ *vs.* E_2 alone analyzed by one way ANOVA followed by Student-Newman-Keuls Method. E_2: estradiol, ORG: Organon2058, ETO: etonogestrol, LNG: levonorgestrel; MPA: medroxyprogesterone acetate DEX: dexamethasone.

Supplementary Figure S2

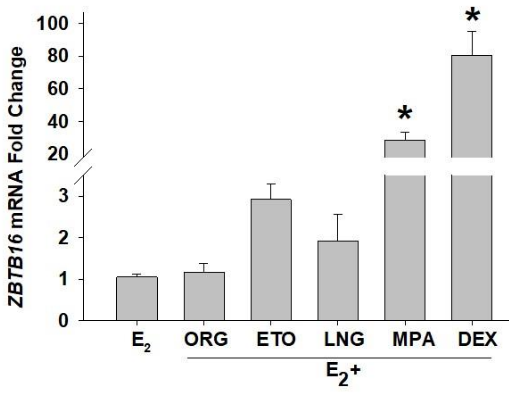

Figure S3. Transient transfection of human *ZBTB16* in primary cultured HESCs. (A) Confirmation of overexpression of *ZBTB16* levels in *ZBTB16* or control vector transfected HESCs treated with or without EMC for 3 days by qPCR. Bars represent mean± SEM;

n=4; *$p<0.05$ *vs*. control vector without EMC; **$P<0.05$ *vs*. Cont-v with EMC treatment analyzed by one way ANOVA followed by Student-Newman-Keuls Method. (**B**) *ZBTB16* mRNA levels in either *ZBTB16* or control vector transfected HESCs treated with or without EMC for 3 days ± 1 U/ml thrombin (THR) for 6 hours. Data represents mean ± SEM; n=4; *$p<0.05$ *vs*. Cont-v or Cont-v + THR. The results were analyzed by one way ANOVA followed by Student-Newman-Keuls Method. Cont-v: Control empty vector; *ZBTB16-v*: *ZBTB16* expressing vector; EMC: E_2+MPA+cAMP.

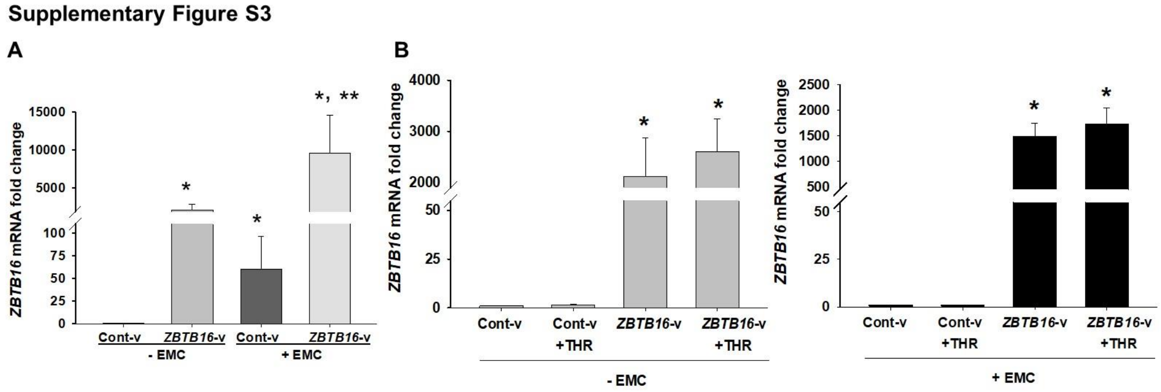

Table S1. Gene list used for this study. Detailed description of primers used for quantitative real time-PCR in this study.

Table S1:

**Gene**	**Full name**	**TaqMan probe ID**
ZBTB16	Zinc finger and BTB domain-containing 16	Hs01561006_m1
PRL	Prolactin	Hs00534909_m1
IGFBP1	Insulin like growth factor 1	Hs01040719_m1
F3	Tissue factor	Hs00765740_m1
ACT	Actin beta	Hs99999903_m1
IL8	Interleukin 8	Hs00199608_m1
PTGS2	prostaglandin-endoperoxide synthase 2	Hs00153133_m1
FKBP5	FKBP proyl isomerase 5	Hs01561006_m1
